# A Proteomic Signature of Healthspan

**DOI:** 10.1093/geroni/igaf122.2087

**Published:** 2025-12-31

**Authors:** Chia-Ling Kuo, Peiran Liu, Richard Fortinsky, George Kuchel, Breno Diniz

**Affiliations:** UCONN HEALTH, Farmington, Connecticut, United States; FDA, Silver Spring, Maryland, United States; University of Connecticut, Farmington, Connecticut, United States; UCONN HEALTH, Farmington, Connecticut, United States; UConn Health, Simsbury, Connecticut, United States

## Abstract

Despite significant efforts to develop biomarkers of aging, few studies have focused on biomarkers of healthspan. We developed a proteomics-based signature of healthspan (healthspan proteomic score (HPS)) using proteomic data from the Olink Explorer 3072 assay in the UK Biobank Pharma Proteomics Project (53,018 individuals and 2920 proteins). Unlike previous biological age predictors trained to predict chronological age, mortality, or organ/disease-specific predictors, HPS was developed to evaluate the risk of a major chronic disease or mortality based on a healthspan definition. HPS outperformed chronological age and other biological age measures in predicting mortality and various chronic diseases. Subgroup analyses revealed that individuals at higher risk for adverse health outcomes—such as males, older adults, current or former smokers, obese individuals, and those with hypertension or hypercholesterolemia—had lower HPS values, indicating a less healthy systemic biological status in these groups. Additionally, the biological processes associated with HPS align with the hallmarks of biological aging, particularly immune response, inflammation, cellular signaling, and metabolic regulation, providing a strong biological validation of HPS as an integrative measure of biological aging. HPS serves as a novel proteomic aging measure, complementing existing proteomic and epigenetic measures. Combining HPS and a proteomic aging clock developed to predict mortality strengthens the associations with mortality and multiple adverse health outcomes. Overall, HPS is a valid biological aging measure, having strong clinical, predictive, and biological validity, making it a valuable tool for assessing healthspan in humans and potentially guiding the development of geroscience-based interventions in the future.

